# How Can Climate Change Anxiety Induce Both Pro-Environmental Behaviours and Eco-Paralysis? The Mediating Role of General Self-Efficacy

**DOI:** 10.3390/ijerph20043085

**Published:** 2023-02-10

**Authors:** Matteo Innocenti, Gabriele Santarelli, Gaia Surya Lombardi, Lorenzo Ciabini, Doris Zjalic, Mattia Di Russo, Chiara Cadeddu

**Affiliations:** 1 Associazione Italiana Ansia da Cambiamento Climatico (AIACC), 50144 Florence, Italy; 2 Section of Hygiene, Department of Life Sciences and Public Health, Università Cattolica del Sacro Cuore, 00168 Rome, Italy; 3 Italian Institute for Planetary Health (IIPH), 00168 Rome, Italy

**Keywords:** eco-anxiety, climate change, adaptation, eco-paralysis, self-efficacy, eco-paralysis, mental health

## Abstract

While it has been shown that climate change anxiety (emotional distress response to climate change) can enhance pro-environmental behaviours (PEBs) in some subjects, in others it can induce eco-paralysis, thus leading individuals to avoid any form of engagement in actions against climate change. This study aims to clarify which factors influence the relationship between climate change anxiety and the disposition to PEBs, focusing on the role of self-efficacy as a mediating factor. A cross-sectional study was conducted on 394 healthy subjects living in Italy who completed the Pro-Environmental Behaviours Scale (PEBS), the General Self-Efficacy scale (GSE), and the Climate Change Anxiety Scale (CCAS). As a result, the mediation model showed a positive direct effect of the cognitive impairment subscale of CCAS on PEBS and an indirect negative effect of the cognitive impairment subscale of CCAS on PEBS mediated by GSE. These findings show that climate change anxiety has simultaneously two different effects on individuals: it directly encourages PEBs, and indirectly may have detrimental effects on PEBs such as eco-paralysis. Consequently, therapeutic approaches to treat climate change anxiety should not be aimed at rationalising irrational thoughts but rather at helping patients develop coping strategies such as PEBs which, in turn, foster self-efficacy.

## 1. Introduction

Climate change is the current global alteration of our planet’s ecological balance, and it represents one of the greatest challenges of the 21st century. According to the *Sixth Assessment Report of Intergovernmental Panel on Climate Change 2022* (IPCC AR6) [1], rising temperatures have led to a global increase in the frequency and intensity of extreme weather events, such as floods, drought, heat waves and hurricanes. Human-induced climate change has already negatively impacted the environment, human health, the economy and society as a whole. Should temperatures exceed 1.5° above pre-industrial levels, the impact of climate change will become even worse.

The health effects of climate change have been extensively reviewed by the Working Group II of IPCC AR6 [1], and classified into: temperature-related effects; premature deaths; direct effects of extreme weather events; consequences of poor air quality; increased risk of infectious diseases; food insecurity and malnutrition; civil conflicts; population displacement and mental health impacts.

Extreme weather events represent difficult stressors to cope with and potential triggers for trauma and mental illness, such as depression and post-traumatic stress disorder [2,3]. Several studies have investigated the relationship between climate change and cognitive responses (e.g., cognitive biases, environmental knowledge, etc.), but recently more importance has been given to emotional involvement [4]. The most studied emotional distress response to climate change is climate change anxiety [5], which is defined by the American Psychological Association as ‘a chronic fear of environmental fate’ [6]. This type of anxiety refers to the anticipation of future problems or potential threats and is associated with worry, rumination, distress, and autonomic and somatic modifications [7]. In this regard, recent studies found higher levels of climate change anxiety in women, who tend to be more concerned about environmental issues than men [8,9,10,11]. According to Susan Clayton, the outcomes of climate change anxiety include cognitive impairment, which affects individuals’ ability to remember, concentrate and make decisions, and functional impairment, which prevents people from conducting certain activities in their daily life [8]. 

To cope with increasingly frequent climate change-related issues, adaptation strategies are required [1]. IPCC AR6 defines adaptation as “the process of adjustment to actual or expected climate and its effects,” and stresses the importance of investigating the range of psychological responses that individuals have towards climate change. In this regard, awareness of climate change is preparatory to concrete social, political and individual interventions, as it drives Pro-Environmental Behaviours (PEBs), defined as behaviours “that a person consciously chooses in order to minimise the negative impact of their actions on the environment” [12,13,14].

As most of the world is aware of the need for global action on climate change, it is pivotal to understand the impact of climate change anxiety on PEBs. The American Psychological Association (2017) argues that negative psychological responses to climate change hamper individuals’ ability to deal with this issue in a constructive manner [6]. More specifically, climate change anxiety may undermine the ability to react, and cause feelings of helplessness and hopelessness [15]. This condition is also known as eco-paralysis [16], which is sometimes misinterpreted as apathy. Eco paralysis is a passive state of behavioural stasis, characterised by depression, excessive anxiety, hopelessness and apathy [16]. By contrast, authors who argue that climate change anxiety encourages people to adopt PEBs assume that PEBs are the most functional adaptive response to climate change anxiety [14]. As stated by Maran and colleagues, “a moderate level of anxiety could engender feelings of virtue, encouraging people to rethink actions with negative ecological impacts [11]”.

Successful adaptation not only depends on governments actions—e.g., disaster preparedness, ecosystem protection, water and waste management—but also on people’s actions that may reduce the negative impact of climate change by adopting climate change adaptive behaviours [17,18] such as PEBs [19]. Prior research has used the theory of planned behaviour [20], norm activation theory [21] and Values–Beliefs–Norms Theory [22] to explain PEBs. However, several authors have also examined PEBs from the perspective of social cognitive theory [23] and, more specifically, personal agency [24], which is defined as a person’s ability to determine and control their actions and life circumstances. One of the mechanisms of personal agency is self-efficacy, [6,25,26] which is people’s beliefs in their ability to control and manage the events and circumstances of their lives, perform specific tasks and obtain desired results. Several studies have shown a positive relationship between self-efficacy and PEBs [9,27,28,29,30,31,32]. According to Bandura [33], having high outcome expectations (i.e., a sense that adopting pro environmental behaviours could be useful in reducing climate change effects) provides motivation during the goal-striving process and influences the way individuals cope with stressors such as climate change-related issues. As stated by Sawitri et al. (2015) [23], self-efficacy fosters positive outcome expectations which, in turn, motivate pro-environmental actions. 

The purpose of this study is to provide deeper insight into the relationship between climate change anxiety and PEBs. The hypothesis made in this study is that self-efficacy is a mediator of the impact of climate change anxiety on PEBs. Climate change anxiety can negatively affect PEB and lead to eco-paralysis via its negative impact on self-efficacy. In other words, we posit a mediation model in which climate change anxiety has positive direct effect on PEBs and negative indirect effect on PEBs via self-efficacy. This study is based on the premises from previous research, which shows a negative correlation between anxiety/climate change anxiety and self-efficacy [30,34,35,36].

While previous research has explored the role of collective self-efficacy in promoting PEBs [37], this paper only considers individual self-efficacy as a mediator in the relationship between climate change anxiety and PEBs. This is because this study is intended to provide therapists with additional insight into the treatment of climate change anxiety. Based on the results of this study, therapists may acquire awareness of the importance of monitoring levels of self-efficacy in patients affected by climate change anxiety, as the ability of these patients to cope with their distress by adopting pro-environmental behaviours is dependent on their self-efficacy beliefs.

## 2. Materials and Methods

### 2.1. Study Design

A mediation analysis was conducted on a cross-sectional sample of healthy participants living in Italy, to determine both the impact of climate change anxiety on PEBs and the role played by self-efficacy in mediating the effects of climate change anxiety on PEBS. 

Power analysis suggested a sample of 191 participants for alpha < 0.05, medium effect size (f = 0.25), Power = 0.80, 2 groups, numerator df = 4, three covariates.

### 2.2. Participants

Participants were selected by adopting a convenience and snowball sampling method, according to three inclusion criteria: age between 18 and 80 years, Italian nationality and being a resident of Italy at the time of the survey. Exclusion criteria were inability to fill out the survey online, and refusal or inability to provide the informed consent. The snowball sampling method was developed as follows: the first participants were selected by sharing the research protocol in the social spaces of the University of Florence. Each participant filled the survey online and was then asked to choose other individuals and send them the questionnaire. The Google Forms platform was used for data collection. Questionnaires for collecting data were administered from February to December 2021. At the bottom of the survey the investigators provided the telephone number and website of the Italian Climate Change Anxiety Association, which offers free psychological help to anyone experiencing the negative effects of climate change through interviews or by providing materials for dealing with ecoanxiety.

The present research study was approved by the local Ethics Committee (Comitato Etico di Area Vasta Centro, CEAVC, of Tuscany), and was conducted according to the criteria defined by the Declaration of Helsinki. Participation was voluntary, and all participants signed an informed consent form before being enrolled in the study.

### 2.3. Instruments

Participants’ levels of climate change anxiety, PEBs and general self-efficacy were assessed once they had completed the Italian version of the Climate Change Anxiety Scale, the Pro-Environmental Behaviours Scale (PEBs Scale) [38], and the General Self-Efficacy scale (GSE scale) [25].

#### 2.3.1. Pro-Environmental Behaviours Scale

The PEBs Scale (PEBS; [38]) consists of a self-administered questionnaire that evaluates how often individuals take actions to reduce or minimise environmental impact in the public and private sectors. It is composed of 19 Likert items with a five point range (from 1 = “never” to 5 = “always”). The Italian version of this scale has valid and reliable psychometric properties. PEBS demonstrated good psychometric characteristics, considering good test–retest reliability (r = 0.85) and good internal consistency (α = 0.80) [27].

#### 2.3.2. General Perceived Self-Efficacy Scale (GSE) 

The GSE Scale (GSE) [25] is a self-reported scale that evaluates the ability of individuals to deal with challenging situations in their everyday life. GSE consists of 10 Likert items with a single factor structure, rated from 1 = “not at all true” to 4 = “exactly true”.

Scholz and colleagues’ assessment of the psychometric properties of the Italian version of the GSE scale showed good validity and acceptable reliability (Cronbach’s α between 0.75 and 0.91) [28]. GSE was used in this study, as it is one of the few scales that has been validated in Italy and has already been successfully used to assess self-efficacy related to climate change in other studies [11]. 

#### 2.3.3. Climate Change Anxiety Scale (CCAS)

The Climate Change Anxiety Scale (CCAS) [8] is a self-report questionnaire generated for measuring levels of climate change anxiety [8,30]. It consists of 13 items with a two-factor structure: cognitive impairment and functional impairment. The cognitive impairment subscale investigates impairment in memory, learning, and concentration caused by thoughts about climate change (“Thinking about climate change makes it difficult for me to concentrate”, “I go away by myself and think about why I feel this way about climate change.”) [30]. The functional impairment subscale investigates impairment in daily activities exacerbated by thoughts about climate change (“My concerns about climate change make it hard for me to have fun with my family or friends.”, “My friends say I think about climate change too much.”) [30]. On a Likert scale from 1 (Never) to 5 (Almost always), participants are asked to indicate how often they are negatively affected by climate change anxiety. The Italian version of this scale has shown valid and reliable psychometric properties, and acceptable internal consistency for both the Cognitive Impairment (α = 0.78) and Functional Impairment subscales (α = 73) [30]. The original version of the CCAS included 22 items and four underlying factors [8]. However, the authors of the Italian validation study suggested that only thirteen items and the first two factors should be used to assess climate change anxiety. This method was used in both the German and Italian validation studies [30,39]. In the Italian validation study, the factor structure for the two-factor model showed poor fitness to data. An exploratory factor analysis suggested that a single factor model could show a better fitness to data, but a confirmatory factor analysis was not conducted [30]. Therefore, in the present study the two-factor model was adopted.

### 2.4. Statistical Analysis

Descriptive statistics were employed to calculate the mean and standard deviations of the continuous variables. Student’s t-test for independent samples was used to assess between-group differences in the assessed variables between male and female participants. Pearson’s partial correlation coefficient was used to assess correlations between variables.

**Figure 1:**
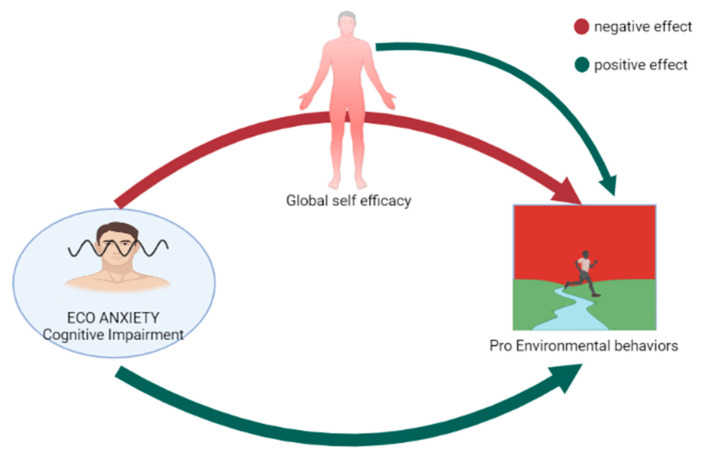
Graphic illustration of the mediation model: general self-efficacy as a mediator of the negative impact of climate change anxiety on pro-environmental behaviours.

A mediation model was used to test the main hypothesis that the negative impact of climate change anxiety on pro-environmental behaviours can be mediated by general self-efficacy. Results were adjusted for sex and age. Mediation analysis examines the pathways by which an independent variable influences a dependent variable via intermediate variables called mediators. In the model tested in the present study, the independent variable was CCAS cognitive impairment subscale, the dependent variable was PEBS, and the mediator was self-efficacy (Figure 1). The model was adjusted for age and sex, because previous studies showed higher levels of climate change anxiety in women, and it was also found that young people are more likely to experience climate change anxiety and engage in PEBs than adults [8,9,10,31]. SPSS software (IBM Corp, IBM SPSS Statistics for Windows, Version 26.0, 2019) was employed for conducting statistical analyses. Power analysis was processed using G*Power 3.1 [32].

## 3. Results

A total of 500 Italian adults (56% female, 44% male, aged 19–76 years) enrolled after providing informed consent. The demographic and socio-economic data collected included age, gender, marital status, level of education, and profession. Out of 500 participants, 394 individuals correctly completed the survey while 106 participants missed at least one response and were excluded from the study. 

The final sample was composed of 394 participants: 141 of them were male and 253 were female, with 281 participants (71.1%) single, and 113 (28.9%) in a stable relationship. Of the total sample, 17 participants (4.3%) had a postgraduate degree, 229 participants (58%) had a bachelor’s degree, 137 participants (35.2%) completed high school, and 11 (2.5%) did not complete high school.

Descriptive analyses for the sample are reported in Table 1.

Age- and sex-adjusted partial correlation coefficients between the analysed variables are shown in Table 2.

A mediation model was used to test the hypothesis that cognitive impairment caused by climate change anxiety can have either a positive direct effect on PEBs or a negative indirect effect on PEBSs by reducing GSE scores. The model was age- and sex-adjusted.

The results of sex- and age-adjusted ANCOVA using the CCAS cognitive impairment subscale as the independent variable and GSE total score as the dependent variable were significant (F(3,390) = 9.739, *p* < 0.001, R2 = 0.06), and the effect of CCAS cognitive impairment on GSE total score was negative and significant (B = −0.324, t = −5.15, *p* < 0.001).

**Figure 2:**
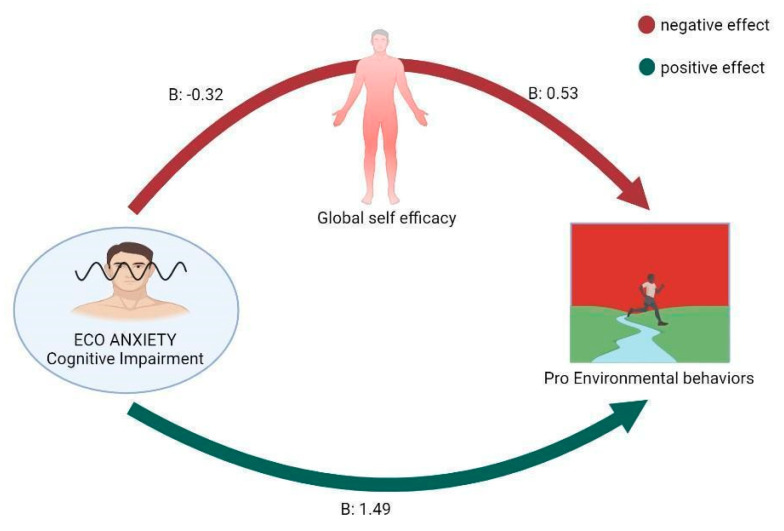
Direct and indirect effect of CCAS on PEBS. (B: regression coefficient).

The results of sex- and age-adjusted ANCOVA using the CCAS cognitive impairment and GSE total score as independent variables and PEBS as the dependent variable was significant (F(4,389) = 33.151, *p* < 0.001, R2 = 0.25). The effect of cognitive impairment related to climate change anxiety on PEBS total scores was positive and significant (B = 1.49, t = 10.90, *p* < 0.001), and the effect of GSE total scores on PEBS total scores was positive and significant (B = 0.53, t = 4.99, *p* < 0.001). Therefore, climate change anxiety had a positive direct effect (direct effect = 1.49) and a significant negative indirect effect (indirect effect: −0.17, Sobel test = −4.48, *p* < 0.001, Bootstrap Standard Error = 0.04, Bootstrap I.C.: −0.30; −0.08) on PEBS mediated by GSE scores (Figure 2).

## 4. Discussion

Climate change is considered one of the greatest challenges of our time, as it is damaging ecosystems and affecting the social and environmental determinants of global health. Effects of climate change could be reduced if people engaged in more pro-environmental behaviours. For this reason, it is crucial to comprehend which factors motivate people to operate in a more eco-conscious way. Alongside ecological values and education, environmental emotions such as ecoanxiety can stimulate pro-environmental behaviours. 

Starting from these premises, our study is the first, to the best of our knowledge, to use a mediation model to analyse the relationship between climate change anxiety and the disposition to PEBs, focusing on the role of self-efficacy as a mediating factor. 

In fact, while previous studies focused on cognitive responses to climate change [2,3,26], this article investigates the relationship between cognitive impairment due to climate change anxiety and PEBs. In this study, levels of climate change anxiety were measured using the cognitive impairment subscale without considering the functional impairment subscale of CCAS. This decision was based on Wells’s cognitive model of generalised anxiety disorder [37], which explains that functional impairment occurs as a result of cognitive impairment. It can be assumed that this also applies to climate change anxiety. In addition, given the low levels of climate change anxiety characterising the individuals examined in this study, it is unlikely that they might have developed functional impairment, which generally occurs at a later stage. 

The main results of this study show that the cognitive impairment arising from climate change anxiety can either enhance or reduce PEBs. This finding leads to the assumption that climate change anxiety has a twofold effect on PEBs. On the one hand, it can stimulate individuals to adopt behaviours that can counteract climate change. This could be interpreted as a coping mechanism [40], which helps individuals affected by the cognitive effects of climate change anxiety to reduce their rumination and worry through the feeling of making a positive impact on the environment. On the other hand, climate change anxiety has a negative impact on general self-efficacy by triggering negative thoughts about global warming and causing a sense of hopelessness and helplessness about the planet’s state. Given that self-efficacy is positively correlated with PEBs, individuals whose self-efficacy is impaired due to climate change anxiety may not be able engage in PEBs [7] and may also experience eco-paralysis [41]. 

The results of our study can have several practical implications: for instance, they suggest that fostering self-efficacy should be the treatment target for individuals with severe climate change anxiety who are unable to develop coping strategies. This is because, while general anxiety is generated by irrational thoughts, climate change anxiety is the result of rational thoughts and fears. For this reason, therapeutic approaches to treat climate change anxiety should not be aimed at rationalising irrational thoughts, but rather at helping patients to develop coping strategies such as PEBs which, in turn, foster self-efficacy. The fundamental role of self-efficacy in the prevention and remediation of climate change anxiety is also supported by studies that show that individual self-efficacy generates collective efficacy. A strong sense of self-efficacy enhances self-esteem, prevents vulnerability and potential mental health disorders, and reduces stress. Moreover, self-perception of ability/competence encourages individuals to take actions that will help them accomplish their goals [7,40]. In this specific context, perceived self-efficacy may lead individuals affected by climate change anxiety to find ways to reduce their negative impact on the environment.

The findings of this study are consistent with previous research by Clayton and Karazsia (2020) [8], Maran [11], and Kellstedt et al. (2008) [42], showing that information about climate change elicits climate change anxiety and motivates people to engage in PEBs by enhancing both individual and collective self-efficacy. Future research on the role of media in increasing self-efficacy and encouraging PEBs might cast additional light on alternative ways to reduce climate change anxiety. 

The results of the present study should be read in the light of some limitations. 

First, the sample showed a lower mean age than the general Italian population, and this could impact the generalizability of the data. 

Second, to the best of our knowledge, no scales have been created thus far to evaluate eco-paralysis; therefore, the possible effect of climate change anxiety on eco-paralysis was inferred by evaluating its relationship with pro-environmental behaviours. 

Third, a transversal design does not allow inferences on the causality of the observed relationship. A longitudinal design should be used to confirm the causal relationship hypothesised in the present study. 

Fourth, no confirmatory factor analysis was made to confirm the two-factor structure of the CCAS, despite some studies suggesting possible alternative factor structures for this scale. 

Finally, while this study focuses on the relationship between individual self-efficacy and PEBs, it could also benefit from taking into account the role of collective self-efficacy.

## 5. Conclusions

The present study provides evidence for a double effect of eco-anxiety on pro-environmental behaviours: a positive direct effect and a negative indirect effect mediated by impairment of self-efficacy. These data could have relevant implications for psychotherapy and mass communication. With regards to therapy, the findings of this study suggest that professionals treating climate change anxiety should also focus on stimulating patients’ self-efficacy to avoid eco-paralysis and encourage pro-environmental behaviours. Future studies on this topic should focus on intervention in order to identify therapeutic and educational strategies that may help increase the adaptive role of eco-anxiety, thus decreasing the risk of eco-paralysis. The development of a well-structured intervention protocol is of crucial importance, especially for young people who are more at risk of being affected by climate change anxiety.

## Figures and Tables

**Table 1:** Descriptive analysis for the study sample.

	Males (N = 141)		Females (N = 253)		Total Sample (N = 394)		*p*-Value
	M	SD	M	SD	M	SD	
**Age**	31.02	10.15	34.35	12.46	33.16	11.78	0.641
**CCAS cognitive impairment score**	11.27	3.68	11.77	3.35	11.59	3.47	0.344
**CCAS cognitive functional impairment score**	6.10	1.91	6.30	1.96	6.23	1.94	0.545
**PEBS total score**	51.56	10.31	50.89	10.65	51.13	10.52	0.231
**GSE total score**	28.80	3.77	28.24	4.81	28.44	4.46	0.07

(M: Mean; SD: Standard Deviation; CCAS: Climate Change Anxiety Scale, PEBS: Pro-Environmental Behaviours Scale, GSE: Global Self-Efficacy).

**Table 2:** Age- and sex-adjusted partial correlation coefficients between the analysed variables are shown.

	M	SD	1	2	3	4
**1.CCAS cognitive impairment**	11.59	3.47	-			
**2.CCAS functional impairment**	6.23	1.94	0.647 ***	-		
**3.PEBS total score**	51.13	10.52	0.440 ***	0.347 ***	-	
**4.GSE total score**	28.44	4.46	−0.253 ***	−0.137 **	0.102 **	-

(M: mean; SD: standard deviation; CCAS: Climate Change Anxiety Scale, PEBS: Pro-Environmental Behaviours Scale, GSE: Global Self-Efficacy ** *p* < 0.01, *** *p* < 0.001; 1, 2, 3, 4 as column headings refer to the different scores shown in the left margin of the table).

## Data Availability

The datasets generated during and/or analysed during the current study are available from the corresponding author on reasonable request.
